# Small-Cell Lung Cancer Presenting as Hypercalcemia

**DOI:** 10.7759/cureus.88367

**Published:** 2025-07-20

**Authors:** Sandra Abadir, Ariel Ahl, Harendra Ipalawatte, Kin Lam, Zain Mehdi

**Affiliations:** 1 Internal Medicine, Los Robles Regional Medical Center, Thousand Oaks, USA

**Keywords:** hypercalcemia, lung cancer, oncologic emergency, parathyroid hormone, small-cell carcinoma

## Abstract

Hypercalcemia of malignancy is a common and life-threatening complication in cancer patients, typically seen in advanced breast cancer, multiple myeloma, and non-small-cell lung cancer. In contrast, it is rare in small-cell lung carcinoma (SCLC), where parathyroid hormone levels remain mostly within the normal range despite elevated calcium. We present a case of hypercalcemia in a patient with SCLC and discuss its clinical implications. A 67-year-old male with a significant smoking history and a history of hypertension and atrial fibrillation presented with symptoms, including nausea, vomiting, fatigue, weight loss, excessive thirst, and pleuritic chest pain. Laboratory tests revealed elevated calcium levels (12.2 mg/dL) and signs of organ involvement. Imaging studies suggested metastatic disease, and a liver biopsy confirmed a metastatic neuroendocrine tumor consistent with SCLC. Further investigation showed suppressed parathyroid hormone and low parathyroid hormone-related peptide levels. Lytic bone lesions were not identified on imaging. Despite hydration therapy, the patient’s hypercalcemia was refractory, and intravenous zoledronic acid was initiated, improving his encephalopathy. He chose comfort measures and passed away several days later. The patient’s hypercalcemia was refractory to hydration, and bisphosphonate therapy was initiated with partial improvement. Despite treatment, the patient’s prognosis remained poor, and he transitioned to palliative care. Hypercalcemia in SCLC, though rare, often indicates a poor prognosis and may be related to mechanisms such as cytokine production or bone marrow involvement. The management of hypercalcemia requires rapid intervention, primarily through intravenous hydration. Bisphosphonates and other adjunct therapies may be necessary for refractory cases. Early palliative care discussions are essential, as these patients typically have a limited life expectancy.

## Introduction

Hypercalcemia is frequently observed in advanced breast cancer, multiple myeloma, and non-small-cell lung cancer, often indicating a poor prognosis [1]. In contrast, it is less commonly seen in small-cell lung carcinoma (SCLC), where parathyroid hormone (PTH) levels typically remain within the normal range despite elevated calcium levels [2]. Hypercalcemia of malignancy is one of the most life-threatening complications of advanced cancer, with patients presenting with a range of symptoms, including nausea, vomiting, abdominal pain, constipation, anorexia, fatigue, anxiety, depression, increased urination, and, in severe cases, arrhythmias and coma [3]. While rare, hypercalcemia can also present as pancreatitis [4].

Patients with hypercalcemia of malignancy generally have a median survival of two to six months from the onset of their disease [3]. Although hypercalcemia is infrequently reported in SCLC, the underlying pathophysiology is not well understood. Some studies suggest that elevated parathyroid hormone-related peptide (PTHrP) levels may play a role [4]. While some believe that bone or bone marrow involvement can contribute to the hypercalcemia, some believe that the condition still remains rare in bone metastasis [4,5].

Regardless of the mechanism behind this metabolic complication, treatment primarily involves intravenous hydration, which may be augmented with loop diuretics, calcitonin, prednisone, denosumab, or hemodialysis [5]. Here, we present the case of a patient who presented with hypercalcemia and was subsequently diagnosed with SCLC.

## Case presentation

A 67-year-old man with a 50-pack-year smoking history, hypertension, and atrial fibrillation on rivaroxaban presented with worsening nausea, vomiting, fatigue, constipation, poor appetite, weight loss, excessive thirst, wheezing, hemoptysis, shortness of breath, pleuritic chest pain, fevers, chills, and night sweats. Vital signs were stable on admission, and physical examination revealed coarse bilateral breath sounds and a diffusely tender, distended abdomen. Laboratory findings are presented in Table 1.

**Table 1:** Relevant laboratory findings along with their reference values.

Laboratory test	Value	Reference value
Calcium	12.2 mg/dL	8.5–10.1 mg/dL
Aspartate aminotransferase	147 IU/L	10–47 IU/L
Alkaline phosphatase	146 IU/L	45–117 IU/L
Carcinoembryonic antigen	6.5 ng/mL	0.0–3.0 ng/mL

**Figure 1:**
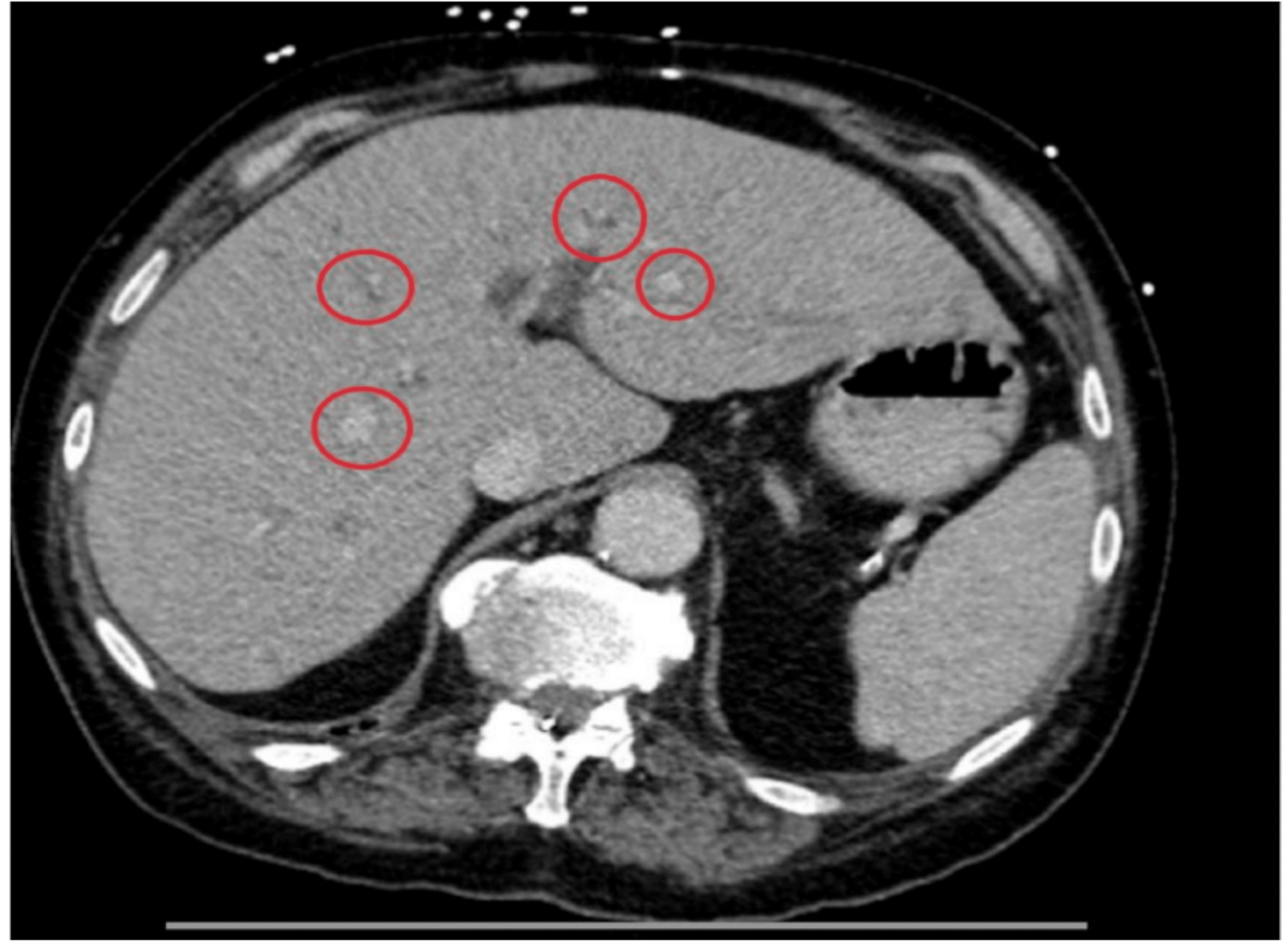
CT of the abdomen and pelvis revealing liver metastatic disease.

A chest X-ray was significant for a left lower lobe pneumonia, prompting initiation of treatment with ceftriaxone and azithromycin. CT of the abdomen and pelvis revealed massive dilation of the rectum, metastatic liver disease (Figure 1), and a left adrenal mass. The initial impression was metastatic colon cancer presenting with large bowel obstruction. The patient underwent exploratory laparoscopy with lysis of sigmoid colon adhesions. Despite palpating the small bowel, rectum, stomach, and pancreas, the primary tumor was not identified. Postoperatively, the patient remained distended, and symptoms minimally improved.

Gastroenterology was consulted for a colonoscopy; however, bowel prep was contraindicated due to significant colonic distention. Further investigations, including lymphoma and leukemia panels, cancer antigen 19-9, alpha-fetoprotein, human chorionic gonadotropin, and 5-hydroxyindoleacetic acid, yielded no significant findings. Lactate dehydrogenase was elevated at 1,377 IU/L (normal range: 85-270 IU/L), and chromogranin levels were also elevated at 745 ng/mL (normal range: 0.0-101.8 ng/mL).

**Figure 2:**
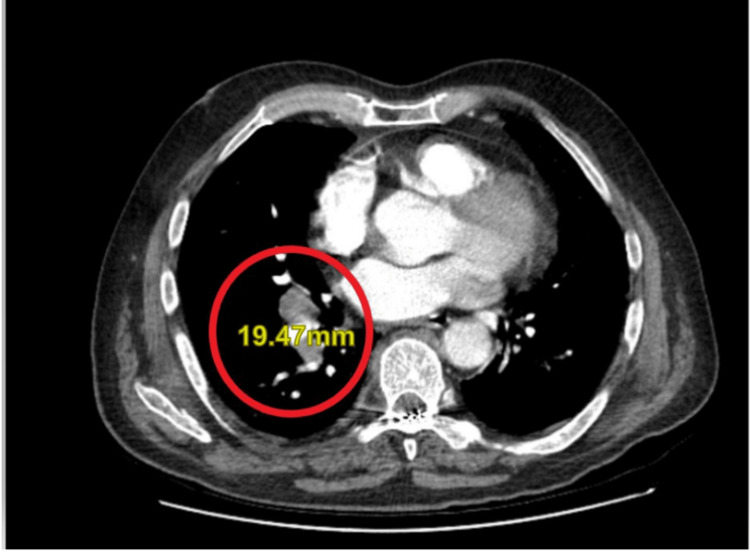
CT angiogram revealing right hilar lymph node measuring 1.9 cm.

A liver biopsy obtained during the procedure revealed a metastatic, poorly differentiated neuroendocrine tumor consistent with SCLC. A CT angiography of the chest showed a right hilar lymph node measuring 1.9 cm (Figure 2), a 1.7 cm nodule in the posterior right base, and patchy opacity in the left base. Pulmonology was consulted for a bronchoscopy with biopsy; however, oncology advised against this approach. The patient chose to pursue outpatient chemotherapy.

The remaining hospital course was complicated by encephalopathy, likely due to a combination of hospital delirium, hypercalcemia, and opioid use. PTH was measured at 6 pg/mL (normal range: 15-65 pg/mL), ionized calcium was 5.9 mg/dL (normal range: 4.65-5.25 mg/dL), PTHrP was <2.0, and vitamin D 25-hydroxy was 23.3 ng/mL (normal range: 30.0-100.0 ng/mL). Lytic bone lesions were not identified on imaging. The patient’s hypercalcemia did not respond to hydration therapy, and he was started on intravenous zoledronic acid, which improved his encephalopathy.

After a goals-of-care discussion with the patient and family, comfort measures were implemented. The patient passed away several days later.

## Discussion

Hypercalcemia is a relatively common complication in cancer patients [4]. It often presents as a paraneoplastic syndrome, most commonly associated with multiple myeloma, leukemias, non-Hodgkin lymphoma, renal cancer, breast cancer, non-small-cell lung cancer, and squamous cell carcinoma of various origins [3,5]. Although rare, hypercalcemia has also been documented in SCLC, even in cases without bone metastasis [4,5]. Approximately 25-30% of cancer patients exhibit hypercalcemia [4,6]. This condition is primarily observed in patients with advanced cancer and is associated with a poor prognosis [5,7].

The exact pathophysiology of hypercalcemia of malignancy remains to be fully elucidated, but several mechanisms have been proposed. One prominent mechanism involves the production of PTHrP [4]. PTHrP acts on the same receptors as PTH, leading to increased serum calcium levels through enhanced production of RANKL, which activates osteoclasts and promotes bone resorption. Additionally, PTHrP can increase serum calcium by enhancing renal calcium reabsorption [5].

Another mechanism is osteolytic metastasis, where excessive calcium is released from bone due to various cytokines, including transforming growth factor-beta, interleukin-6, interleukin-3, and RANKL. This mechanism is most commonly observed in multiple myeloma and breast cancer. A third proposed mechanism involves ectopic activity of 1-alpha-hydroxylase, leading to the production of 1,25-dihydroxyvitamin D. Finally, ectopically produced PTH may also contribute to hypercalcemia. In patients with SCLC, one hypothesis suggests that hypercalcemia may result from bone marrow involvement, leading to cytokine production, osteoclast activation, and subsequent hypercalcemia [5].

In our case, the usual mechanisms of hypercalcemia of malignancy were not applicable. Our patient did not have elevated PTH or PTHrP levels, and vitamin D levels were low, which is atypical for hypercalcemia associated with malignancy. Additionally, no osteolytic lesions were observed. These findings make our case unusual, as most cases of hypercalcemia in cancer patients can be explained by mechanisms such as PTHrP production or osteolytic metastasis, as seen in multiple myeloma and breast cancer [4,5]. While there is some evidence to suggest that hypercalcemia in SCLC can be linked to bone marrow involvement, cytokine production, and osteoclast activation, these mechanisms were not observed in our patient [5]. Our case could contribute to the understanding that hypercalcemia in SCLC may, in rare instances, not follow the conventional pathophysiologic pathways, raising questions about alternative or previously unrecognized mechanisms. This may also have implications for diagnostic and therapeutic approaches in similar cases of hypercalcemia where typical markers and mechanisms are absent.

The management of hypercalcemia depends on the patient’s symptoms and the magnitude of calcium elevation. Patients with hypercalcemia of malignancy are often symptomatic with significantly elevated calcium levels, necessitating rapid intervention as this condition is considered an oncologic emergency. Additionally, because most cancer patients presenting with hypercalcemia have a poor prognosis, discussions regarding goals of care should be prioritized, and a palliative care team should be involved.

The first step in treatment is intravenous hydration, preferably using isotonic crystalloid solutions such as normal saline. Patients should be periodically assessed for signs and symptoms of fluid overload. Loop diuretics are generally avoided due to the risk of dehydration, except in cases where fluid overload develops. In patients with cardiorenal disease and refractory severe hypercalcemia who cannot be safely hydrated, hemodialysis should be considered [7].

For patients with severe hypercalcemia, particularly in the context of malignancy, intravenous hydration alone is usually insufficient. The next step in management involves the use of calcitonin, which can be administered every 6-12 hours and may reduce calcium levels by approximately 2 mg/dL. However, its use is limited to 48 hours due to decreased efficacy and potential tachyphylaxis [7].

Bisphosphonates are a key component of therapy, particularly in cases of hypercalcemia of malignancy, with zoledronic acid being the most potent option. Caution is warranted in patients with underlying renal disease. For cases refractory to bisphosphonates or in patients with renal impairment where bisphosphonates are contraindicated, denosumab may be considered [7-9]. In patients with lymphoma, the addition of prednisone can help reduce gastrointestinal absorption of calcium [7].

## Conclusions

While hypercalcemia is commonly associated with many malignancies, it is considered uncommon in SCLC. Nevertheless, it is linked to a poor prognosis and necessitates rapid and aggressive treatment to alleviate symptoms. Hydration remains the cornerstone of management; however, some patients may require additional therapies such as bisphosphonates, prednisone, calcitonin, or hemodialysis if they do not respond adequately to intravenous fluids.
